# Molecular Docking Analysis of Heparin–Diclofenac Complexes: Insights into Enhanced Cox Enzyme Inhibition for Pain Management

**DOI:** 10.3390/life15121903

**Published:** 2025-12-12

**Authors:** Manuel Ovidiu Amzoiu, Oana Taisescu, Emilia Amzoiu, Andrei Gresita, Georgeta Sofia Popescu, Gabriela Rău, Maria Viorica Ciocîlteu, Costel Valentin Manda

**Affiliations:** 1 Faculty of Pharmacy, University of Medicine and Pharmacy of Craiova, 200638 Craiova, Romania; manuel.amzoiu@umfcv.ro (M.O.A.); gabriela.rau@umfcv.ro (G.R.); maria.ciocilteu@umfcv.ro (M.V.C.); valentin.manda@umfcv.ro (C.V.M.); 2 Faculty of Medicine, University of Medicine and Pharmacy of Craiova, 200638 Craiova, Romania; oana.taisescu@umfcv.ro (O.T.); andrei.gresita@umfcv.ro (A.G.); 3 Faculty of Food Eng, University of Life Science “King Michael” from Timisoara, 300645 Timisoara, Romania; sofiapopescu@usvt.ro

**Keywords:** heparin, diclofenac, COX-1, COX-2, supramolecular complexes, molecular docking, dual-drug docking, molecular dynamics, lipophilicity

## Abstract

The aim of this study was to investigate the molecular interactions of heparin, diclofenac, and their supramolecular complexes with cyclooxygenase enzymes (COX-1 and COX-2) using computational docking techniques. Diclofenac is a widely used nonsteroidal anti-inflammatory drug (NSAID) that inhibits COX isoforms, whereas heparin is a polyanionic glycosaminoglycan with established anticoagulant and emerging anti-inflammatory properties. Supramolecular association between these agents may modulate their physicochemical behavior and target engagement. Molecular modeling, dual-drug docking, and molecular dynamics (MD) simulations were employed to characterize the interactions of heparin, diclofenac, and pre-formed heparin–diclofenac complexes with COX-1 and COX-2. Geometry optimization and lipophilicity (logP) estimates were obtained using HyperChem, while protein–ligand docking was performed in HEX using crystallographic COX structures from the Protein Data Bank. Docking poses were analyzed in Chimera, and selected complexes were refined through short MD simulations. Pre-formed heparin–diclofenac assemblies exhibited markedly enhanced docking scores toward both COX isoforms compared with single ligands. Binding orientation strongly influenced affinity: for COX-1, the heparin–diclofenac configuration yielded the most favorable interaction, whereas for COX-2 the diclofenac–heparin configuration was preferred. Both assemblies adopted binding modes distinct from free diclofenac, suggesting cooperative electrostatic and hydrophobic contacts at the enzyme surface. Supramolecular complexation also altered calculated logP values relative to the individual compounds. MD simulations supported the relative stability of the top-ranked complex–COX assemblies. These findings indicate that heparin–diclofenac assemblies may enhance and reorganize predicted COX interactions in a configuration-dependent manner and illustrate the utility of dual-drug docking for modeling potential synergistic effects. Such insights may inform the design of localized or topical formulations, potentially incorporating non-anticoagulant heparin derivatives, to achieve effective COX inhibition with reduced systemic exposure. However, the results rely on simplified heparin fragments, legacy docking tools, and short MD simulations, and should therefore be interpreted qualitatively. Experimental studies will be essential to confirm whether such supramolecular assemblies form under physiological conditions and whether they influence COX inhibition in vivo.

## 1. Introduction

Heparin is a naturally occurring anticoagulant widely used to prevent and treat thromboembolic disorders such as deep vein thrombosis (DVT), pulmonary embolism (PE), and myocardial infarction (MI). It is derived from animal tissues, primarily porcine intestinal mucosa or bovine lung, and exists in two main forms: unfractionated heparin (UFH) and low molecular weight heparin (LMWH). Both forms enhance the activity of antithrombin III, a plasma protein that inhibits thrombin and factor Xa, thereby disrupting the clotting cascade and preventing fibrin clot formation [1]. UFH is a heterogeneous mixture of polysaccharides with varying molecular weights, conferring rapid onset but requiring close monitoring due to an unpredictable dose–response relationship and the risk of heparin-induced thrombocytopenia (HIT) [1]. LMWH exhibits more predictable pharmacokinetics, greater bioavailability, and a lower incidence of HIT, making it suitable for outpatient use [2].

Beyond anticoagulation, heparin has been increasingly recognized for its anti-inflammatory, antiviral, and anticancer properties, mediated through interactions with chemokines, cytokines, and growth factors [3]. Several groups have also explored supramolecular assemblies and dual-drug strategies to enhance NSAID delivery and modulate COX inhibition, including carrier-based naproxen and ibuprofen formulations and multi-ligand docking protocols aimed at capturing cooperative binding effects. These emerging approaches suggest that the physicochemical plasticity of heparin makes it a suitable scaffold for supramolecular interaction studies.

Diclofenac is a widely used nonsteroidal anti-inflammatory drug with analgesic, anti-inflammatory, and antipyretic properties. It is indicated for pain and inflammation associated with osteoarthritis, rheumatoid arthritis, ankylosing spondylitis, and musculoskeletal injuries. Diclofenac inhibits the cyclooxygenase enzymes COX-1 and COX-2, which mediate prostaglandin synthesis involved in inflammation, pain, and fever [4]. Its diverse formulations—including oral tablets, topical gels, transdermal patches, and injectables—enable management of both acute and chronic pain, with topical delivery offering localized analgesia and reduced systemic exposure [5]. Despite its efficacy, diclofenac carries gastrointestinal, cardiovascular, and renal risks, especially with prolonged or high-dose use [6]. It is also effective in postoperative pain and dysmenorrhea, with rapid onset and well-characterized pharmacokinetics [7].

Diclofenac was selected for this proof-of-concept study because it is extensively characterized with respect to COX binding and inhibition, is widely used in topical and localized formulations—consistent with the mechanistic aims of this work—and has been previously examined in our dual-drug docking studies with heparin and factor Xa [8]. We acknowledge that diclofenac carries a higher cardiovascular risk than NSAIDs such as naproxen or ibuprofen; thus, its selection reflects mechanistic familiarity rather than an optimal clinical safety profile.

Although heparin and diclofenac act through different mechanisms, both agents can influence inflammatory pathways. Heparin may alleviate pain under inflammatory or thromboembolic conditions by reducing pro-inflammatory mediator activity [3], whereas diclofenac suppresses prostaglandin synthesis via COX inhibition [9]. Their availability in multiple formulations, including oral, topical, and injectable preparations, allows individualized treatment strategies, with topical diclofenac offering a favorable safety profile [10,11]. These complementary pharmacological properties motivate exploration of possible cooperative interactions between the two molecules at the receptor level.

Molecular docking is a computational technique that predicts the preferred orientation of a ligand when binding to a macromolecular target, enabling insight into binding modes, virtual screening, and structure-based drug design [12,13]. Traditional docking, however, typically considers a single ligand interacting with a receptor and therefore cannot capture synergistic or cooperative interactions between multiple ligands. Dual-ligand docking approaches have been proposed to model multi-component systems and supramolecular drug assemblies [8]. In this context, the present study aims to characterize, in silico, the interactions of pre-formed heparin–diclofenac supramolecular complexes with COX-1 and COX-2, and to compare their predicted binding behavior with that of the individual ligands.

Despite its advantages, molecular docking has inherent limitations. Prediction accuracy depends on receptor quality, ligand and protein flexibility, and the scoring algorithm used [12]. Furthermore, supramolecular systems involving large polyanions pose unique methodological challenges that remain incompletely addressed by standard docking algorithms. The use of a 2-ring heparin fragment in this study represents a major simplification: real heparin is substantially larger, highly heterogeneous, and conformationally dynamic. Similarly, the molecular dynamics simulations employed here—conducted on a picosecond timescale—are far shorter than the 300–500 ns typically required for robust biomolecular dynamics and therefore provide qualitative rather than quantitative insights into stability and binding kinetics.

In this context, the present study introduces an integrated supramolecular docking and simulation strategy that extends beyond classical single-ligand approaches. First, we generate pre-formed heparin–diclofenac supramolecular complexes and treat them as dual-drug ligands to examine how complexation reshapes the pharmacophore presented to the COX active site. Second, we systematically compare the interactions of these complexes with both COX-1 and COX-2 to identify isoform-specific preferences. Third, we complement docking with molecular dynamics simulations in explicit solvent to evaluate short-timescale stability under near-physiological conditions. Together, these steps provide a mechanistic framework for assessing whether supramolecular association could theoretically reorganize COX binding interactions and inspire future experimental work.

## 2. Materials and Methods

### 2.1. HyperChem Software

We conducted a chemical modeling study on the molecules heparin and diclofenac using the HyperChem program [14]. The 2D models of the studied compounds were prepared using the HyperChem software and subsequently subjected to geometric optimization through two different protocols. The first protocol utilized the MM+ (Molecular Mechanics Force Field), while the second applied the semi-empirical PM3 force field, both implemented in HyperChem (v 8.0.8, HyperCube, Gainesville, FL, USA) [14]. To achieve optimization, we employed the Polak–Ribiere conjugate gradient algorithm with an RMS gradient threshold of 0.1 kcal/(Å·mol). Among the resulting conformations, we selected the most stable variants for further analysis. Furthermore, the theoretical logP value, an important descriptor of molecular lipophilicity, was calculated using the QSAR (Quantitative Structure–Activity Relationship) module of HyperChem [14]. This parameter provides insight into the compound’s ability to interact with biological membranes and its potential pharmacokinetic properties.

### 2.2. HEX Software

The binding interactions of these molecules with the active site of the receptor were analyzed using HEX software, version 8.0.0 [15]. A standard docking protocol was implemented, selecting the “Shape + Electro” correlation mode to account for both steric and electrostatic complementarity. In the initial docking stage, diclofenac and heparin were each designated as both receptor and ligand to generate supramolecular assemblies. To construct these complexes, diclofenac and heparin were docked against one another in HEX using the same “Shape + Electro” correlation mode [14]. In one configuration, diclofenac served as the ligand and heparin as the receptor (diclofenac_heparin), whereas in the reciprocal configuration, heparin was the ligand and diclofenac the receptor (heparin_diclofenac). For each orientation, the top 100 docking poses were generated and ranked by docking energy. The most stable pose exhibiting a physically plausible orientation specifically, diclofenac positioned proximal to the ether oxygen bridging the two heparin rings and oriented toward available sulfate groups was selected as the representative supramolecular complex. This non-covalent assembly was subsequently exported and used as a dual-drug ligand in docking simulations with COX-1 and COX-2 (Supporting Information).

To ensure accurate docking calculations, the HEX tool automatically removed all crystallographic water molecules and other heteroatoms from the input structures. It then reoriented each protein around its coordinate origin and evaluated the separation between the molecular origins as part of the docking procedure. For each configuration, docking scores were computed, and the highest-ranking orientations were retained. Following docking, the top 100 conformations were collected, and the most stable structures—based on docking energies and steric plausibility—were selected for detailed inspection. The receptor structures used in this study were obtained from the Protein Data Bank (PDB) [16].

To assess the influence of hydrophobicity, or lipophilicity, on ligand behavior [17,18], partition coefficient (logP) values for diclofenac and heparin were calculated using the HyperChem program [14] (Table 1). Docking simulations were conducted using heparin fragments composed of 2, 4, 6, and 8 saccharide rings. Structural analysis revealed that in the 4-, 6-, and 8-ring models, diclofenac consistently localized near the ether oxygen positioned immediately after the second heterocyclic unit. The recurrence of this motif across multiple fragment lengths indicated that the essential interaction pattern is captured within a shorter heparin segment. Consequently, the 2-ring heparin model was selected as the representative structure, as it preserves the critical structural region—two heterocycles bridged by an ether oxygen—while improving computational tractability and interpretability.

We emphasize that this truncation represents a strong simplification of full-length heparin, which is a heterogeneous, highly charged polysaccharide with extensive conformational flexibility. Thus, the results presented here should be interpreted as qualitative, local interaction models rather than as complete representations of the entire polymer.

The computational tools employed in this study (HyperChem and HEX) are legacy platforms and are not optimized for large, highly anionic glycosaminoglycans. Consequently, all docking energies and MD trajectories should be regarded as qualitative indicators of relative trends rather than quantitative predictions of binding free energies, kinetic behavior, or long-timescale dynamics.

### 2.3. Molecular Dynamics and Simulation Protocol

The initial molecular structure was prepared in HyperChem (version 8.0) [14] by entering selection mode and setting the selection level to atoms. The nitrogen atom was selected and assigned a charge of +1 via the Set Charge function in the Build menu. After clearing the selection (right-click in an empty space), both oxygen atoms were selected and assigned a charge of −0.5 each. For energy minimization, Molecular Mechanics was selected from the Setup menu, and the AMBER force field was chosen from the Molecular Mechanics Force Field dialog. Additional parameters were set under the Options menu.

To solvate the system, a periodic water box was generated using the Periodic Box function in the Setup menu. A rectangular box of pre-equilibrated water molecules was defined, with the solute placed at the center and a minimum distance specified between solute and solvent atoms. Water molecules that overlapped (i.e., those with atoms closer than the defined distance to solute atoms) were automatically removed by the software. Geometry optimization was performed using the Compute > Geometry Optimization option, including all solvent molecules. If convergence was not achieved, the maximum cycle count was increased to 800 within the Molecular Mechanics settings, and the optimization was repeated.

For molecular dynamics simulations, the Molecular Dynamics option under the Compute menu was used. The MD simulation followed a simulated annealing protocol, consisting of three phases: heat, run, and cool. During the heating phase, the system was brought from an initial temperature of 100 K to the target temperature of 300 K using velocity rescaling. The heat time was set to 0.1 picoseconds, and the temperature step was set to 30 K. The run phase followed, with the system simulated at constant temperature using velocity rescaling. A run time of 0.5 picoseconds was used. An optional cooling phase could be added by decreasing the temperature in defined increments.

For each MD simulation, relevant averages were selected, including EKIN (kinetic energy), EPOT (potential energy), ETOT (total energy), and TEMP (temperature), allowing for monitoring of simulation stability and energy changes.

Short MD runs (total simulation time in the picosecond range) were performed in HyperChem using the AMBER force field, with the primary aim of locally relaxing the docking poses in explicit water rather than sampling long-time dynamics. These brief simulations serve as an additional consistency check for the relative stability ordering suggested by docking, but they do not constitute a rigorous 300–500 ns biomolecular simulation.

The graphical representations of the molecular dynamics simulation results were created using R software (version 4.5) [18]. The numerical data used for plotting were first processed and organized in Microsoft Excel, then imported into R software for visualization and analysis.

## 3. Results

The initial phase of our study utilized the HyperChem program to conduct molecular modeling of compounds heparin and diclofenac. Following this, Hex 8.0.0 software was employed to assemble these compounds into complexes. In the docking simulations, one compound was designated as the ligand, while the other acted as the receptor. The primary objective of this analysis was to evaluate whether the binding sequence of these two compounds within the complex affected the results (see Table 2).

The docking results presented in Table 2 reveal different binding energies of the diclofenac–heparin and heparin–diclofenac complexes, highlighting the influence of the ligand–receptor designation on binding interactions. The energy values, expressed in negative terms, represent the strength of the binding affinity, with lower values indicating stronger interactions. When diclofenac was designated as the ligand and heparin as the receptor, the binding energy was calculated as −140.56. Conversely, when the roles were reversed—heparin as the ligand and diclofenac as the receptor—the energy was slightly lower at −146.73, indicating a stronger binding affinity. This difference suggests that the orientation and specific interactions between the two molecules depend on their assigned roles during the docking process. The stronger interaction in the heparin-as-ligand scenario could be attributed to its larger and more flexible structure compared to diclofenac. Heparin, being a polysaccharide, may have more opportunities for favorable interactions such as hydrogen, ionic, or van der Waals interactions when actively searching for binding sites on diclofenac. On the other hand, the smaller and relatively rigid structure of diclofenac may limit its ability to fully optimize interactions when designated as the ligand. The Hex docking program employs a rigid receptor model, which assumes that the receptor remains static during the docking process, while the ligand retains flexibility to explore different binding conformations [15]. The spatial configuration of the resulting complexes can vary depending on whether the ligand is docked first or second, particularly in cases involving multiple ligands.

To address this limitation, we incorporated both spatial configurations of the complexes into our docking process: (A) where diclofenac is docked first and heparin second, and (B) where heparin is docked first and diclofenac second. This dual-configuration approach allows us to account for the potential structural variability introduced by ligand binding and ensures a more comprehensive analysis of the synergistic interactions between the two molecules. By considering both configurations, we aim to better understand the molecular dynamics and improve the predictive accuracy of the docking process for such multi-ligand systems.

The conformational analysis of the studied complexes reveals key differences between them, primarily through variations in bond angles between the atoms within each molecule and the intermolecular distances obtained from the docking process (Table 3). These structural variations can significantly influence the stability and binding affinity of the complexes, ultimately affecting their biological interactions.

The determination of these conformational parameters was made possible after optimizing the molecular structures using the HyperChem software [14]. This optimization step allowed for a more accurate representation of the molecular geometry, ensuring that bond angles and distances were refined before further analysis. By applying this method, we were able to better understand the spatial arrangement of the complexes and their potential implications in receptor binding and molecular interactions.

The theoretical data presented in this table provide a solid foundation for determining the geometry of the studied complexes, reinforcing the conformational differences observed during the docking process of the two compounds, heparin and diclofenac. These differences in molecular conformation are essential for understanding how the complexes interact with their respective receptors, influencing their binding stability and overall biological activity.

Furthermore, the variations in physicochemical parameters, determined using HyperChem, highlight distinct structural and electronic properties of the studied molecules [14]. These values, which include bond angles, intermolecular distances, and other molecular descriptors, offer valuable insights into the behavior of the complexes and their potential functional implications. The specific values of these parameters are detailed in Table 4. This analysis underscores the importance of considering conformational flexibility and spatial arrangement when evaluating molecular interactions, as even small structural variations can significantly impact docking results and subsequent biological effects.

As observed in Table 4, variations occur in all these parameters, supporting the differences in conformations presented by the two studied complexes. Among these, SA and V are molecular shape descriptors, while the electric dipole moment is a measure of the polarization of the molecular system. This observation underscores the importance of exploring different ligand–receptor configurations in molecular docking studies to ensure a comprehensive understanding of binding interactions. Such differences in binding energy can guide the design of experiments or therapeutic strategies, particularly when considering the implications of ligand–receptor orientation in drug–receptor dynamics. Further analysis, including the examination of specific interaction sites and the contribution of hydrophobic and electrostatic forces, could provide deeper insights into the binding mechanisms. A key factor in understanding these interactions is lipophilicity, typically expressed as the logarithm of the partition coefficient (logP), which measures the affinity of a compound for lipid (or octanol) phases compared to water [17]. LogP values are crucial for predicting solubility, permeability, and overall bioavailability of a molecule. In this study, we evaluate values of logP to examine their influence on the interactions within the diclofenac–heparin complexes.

Table 5 presents the calculated logP values for the diclofenac–heparin and heparin–diclofenac complexes, providing insight into the lipophilicity of these systems and its potential impact on their behavior in biological environments. The logP value reflects the preference of a compound or complex for lipid phases over aqueous phases, with higher values indicating greater lipophilicity.

In the heparin–diclofenac complex, the value of logP is 4.04, slightly higher than 3.76 observed for the diclofenac–heparin complex. This difference suggests that the orientation and role of each molecule in the complex influence the overall hydrophobicity. The higher logP value for the heparin–diclofenac configuration indicates that this arrangement is more lipophilic, which could enhance its interaction with lipid membranes or hydrophobic environments. This increased lipophilicity might be due to the alignment or exposure of more hydrophobic regions of the molecules in this particular orientation.

In contrast, the diclofenac–heparin complex exhibits a lower logP value, implying it is slightly more hydrophilic than the reverse configuration. This could reflect differences in the exposure of polar functional groups or variations in the overall packing and interaction between the molecules when diclofenac acts as the ligand.

These findings highlight the importance of lipophilicity in determining the behavior of molecular complexes. A more lipophilic configuration might enhance membrane permeability and retention in lipid-rich environments, while a more hydrophilic orientation could favor interactions in aqueous media. Such differences can have implications for drug delivery and bioavailability, emphasizing the need to consider lipophilicity when designing and evaluating drug–receptor complexes [19]. Further experimental validation and analysis of specific interaction sites could clarify the role of these logP differences in biological settings.

In the next phase of our study, we present the results of molecular docking simulations involving our complexes and the Protein Data Bank (PDB) receptors 3N8V (COX-1) [20] and 5W58 (COX-2) [21]. Cyclooxygenase (COX) enzymes are pivotal in pain management, as they mediate the production of prostaglandins involved in inflammation and pain. By incorporating structural data, this analysis aims to reveal how our complexes interact with these enzymes, providing insights into the three-dimensional nature of these interactions and their potential pharmacological relevance [16].

The data in Table 6 present the binding energies of various configurations involving heparin and diclofenac, either as individual compounds or as complexes. The complex heparin_diclofenac shows the most favorable binding energy at −358.06 kcal/mol, significantly lower than the energies observed for heparin alone (−309.55 kcal/mol) and diclofenac alone (−305.47 kcal/mol). This indicates that when heparin and diclofenac are combined in this specific complex, they form a stable interaction with enhanced binding affinity compared to either compound taken alone.

In contrast, the alternative complex configuration, diclofenac_heparin, exhibits a much higher binding energy of −63.7 kcal/mol, suggesting a far weaker interaction. This discrepancy between the two complexes indicates that the sequence in which heparin and diclofenac bind is critical to their overall stability. The heparin_diclofenac complex, where heparin likely acts as the receptor and diclofenac as the ligand, appears to enable stronger interactions, possibly due to optimal alignment of hydrophobic and electrostatic interactions.

The marked difference in binding energies between the two complexes underscores the significance of binding orientation and order. The high stability configuration of the heparin_diclofenac complex may enhance the pharmacological potential of this complex, as stronger binding can often correlate with better target specificity and bioactivity [22]. These findings could provide a basis for future studies exploring the structural basis of binding preferences and optimizing the sequence of binding for improved therapeutic efficacy. Further structural analyses, such as molecular dynamics simulations, may help clarify the interaction mechanisms and identify the specific molecular interactions responsible for this increased stability.

The docking results presented in Table 7 display the binding energies of different configurations involving diclofenac, heparin, and their complexes with COX-2 enzyme. The complex diclofenac_heparin exhibits the lowest binding energy at −468.48 kcal/mol, indicating the most stable interaction with COX-2. This high stability suggests that, when diclofenac acts as the ligand binding to heparin within the complex, the resulting structure has an enhanced affinity for COX-2 active site.

The alternative complex, heparin_diclofenac, shows a slightly higher binding energy of −434.59 kcal/mol. Although still a stable interaction, it is weaker compared to the diclofenac_heparin configuration, implying that the order in which these compounds bind affects the strength of the interaction with COX-2. The binding energies of the individual compounds, with diclofenac at −332.81 kcal/mol and heparin at −285.75 kcal/mol, are notably higher, indicating less stable interactions with COX-2 compared to the complexes. This suggests that the formation of a complex between diclofenac and heparin significantly enhances their collective binding affinity for COX-2, likely due to combined molecular interactions that are not achievable by either compound alone.

The results underscore the importance of binding orientation and sequence in complex formation, as it directly impacts binding strength with COX-2. The stronger binding affinity observed in the diclofenac_heparin complex may translate to improved inhibitory effects on COX-2, which could be advantageous in therapeutic contexts where COX-2 inhibition is desired. These findings suggest potential benefits in exploring diclofenac–heparin combinations for enhanced COX-2 targeting [23]. Further studies, such as molecular dynamics simulations or experimental validation, could help clarify the specific interactions that contribute to this stability and optimize the design of diclofenac–heparin-based complexes for COX-2 inhibition.

**Figure 1:**
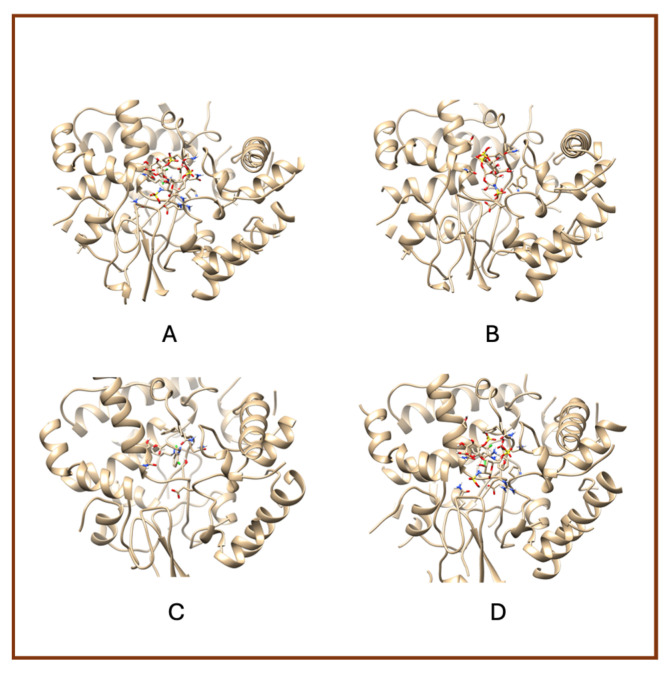
High-resolution 3D docking images of the COX-1 receptor with (**A**) heparin_diclofenac complex, (**B**) heparin, (**C**) diclofenac, (**D**) diclofenac_heparin complex These representations highlight the spatial arrangement and major contact regions of each ligand within the COX-1 active site [24].

A notable observation from the docking images is that the heparin–diclofenac and diclofenac–heparin complexes bind at a site distinct from the binding sites of the individual drugs, heparin and diclofenac (Figure 1). This difference in binding location may reflect altered spatial and chemical properties of the complexes compared to the individual molecules.

Furthermore, the docking results suggest that the strength of binding varies between the two complexes. Specifically, the diclofenac–heparin complex appears to exhibit weaker binding compared to the heparin–diclofenac complex, as indicated by the binding energy calculations. This disparity could be attributed to differences in the orientation and accessibility of key functional groups within each complex, which influence their interactions with COX-1 receptor. For instance, the heparin–diclofenac complex may align more favorably, allowing for stronger interactions with amino acid residues of the receptor, while the diclofenac–heparin complex might have a less optimal conformation.

**Figure 2:**
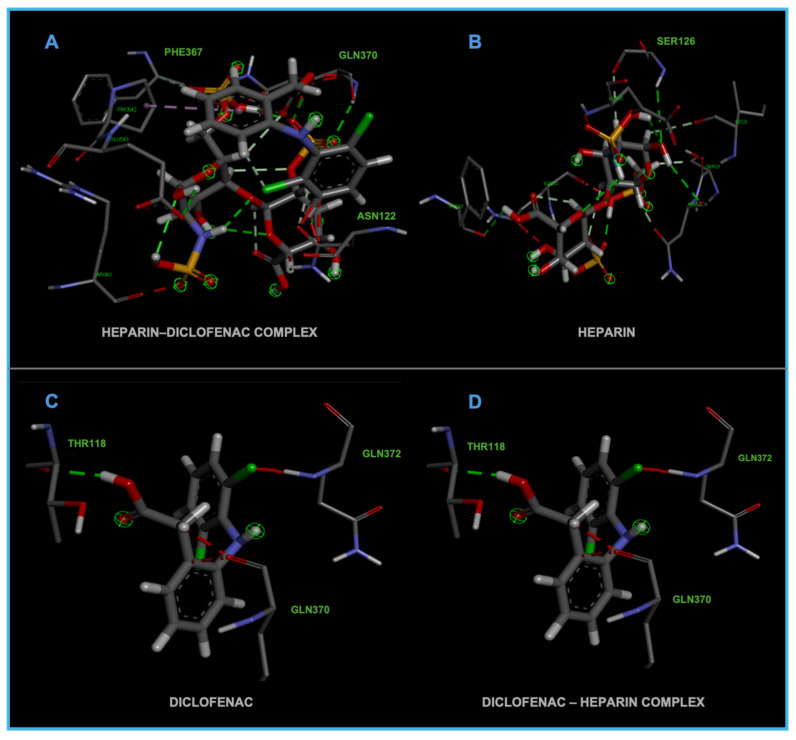
High-resolution 3D docking images of the COX-1 receptor with (**A**) heparin–diclofenac complex, (**B**) heparin, (**C**) diclofenac, and (**D**) diclofenac–heparin complex. These representations highlight the spatial orientation of each ligand within the COX-1 active site and the key interacting amino acids involved in binding [25].

The distinct binding sites and varying binding strengths highlight the importance of ligand–receptor orientation and molecular structure in determining the efficacy of drug–receptor interactions (Figure 2). These findings could have implications for designing combination therapies or conjugate drugs, as the binding behavior of complexes differs significantly from that of their constituent drugs.

The distinct interaction patterns of the four tested substances within the COX-1 binding site emphasize how structural differences influence their affinity and mode of binding. The heparin–diclofenac complex (A) demonstrates a stable interaction profile, anchoring to essential residues such as Arg120 and Tyr355, which are well-known contributors to COX-1 ligand recognition. This suggests that the conjugated form may enhance binding stability compared to individual components (Figure 2). In contrast, heparin alone (B) interacts predominantly through electrostatic contacts, particularly with positively charged amino acids including Arg120 and Lys83. While these interactions confirm heparin’s ability to occupy the vicinity of the active site, they lack the hydrophobic contributions typically associated with strong COX-1 inhibitors. Diclofenac (C) shows the expected binding pattern characteristic of classical NSAID inhibitors, engaging both Arg120 and Tyr355 while stabilizing within the hydrophobic pocket of the catalytic site. This confirms the reliability of the docking protocol and reflects diclofenac’s well-described inhibitory mechanism. The diclofenac–heparin complex (D) presents a hybrid interaction profile that combines the electrostatic features of heparin with the hydrophobic and anchoring residues required for diclofenac activity. The co-engagement of Arg120, Tyr355, and nearby hydrophobic residues suggests that conjugation may modulate ligand orientation within the active site, potentially affecting both binding affinity and functional outcome.

Overall, these interaction patterns highlight how molecular combination or conjugation can influence COX-1 binding behavior, offering insights into potential modifications that may alter efficacy, selectivity, or safety in pharmacological applications.

**Figure 3:**
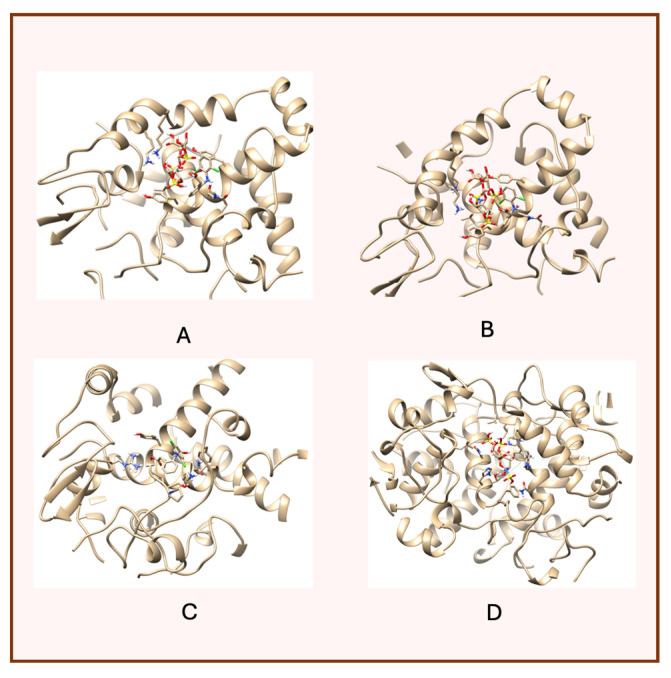
High-resolution 3D docking images of the COX-2 receptor with (**A**) heparin_diclofenac complex, (**B**) diclofenac_heparin complex, (**C**) diclofenac, (**D**) heparin. These images illustrate the ligand orientations and principal interaction zones within the more flexible catalytic pocket of COX-2 [24].

A notable finding from these docking simulations is that both complexes—heparin–diclofenac and diclofenac–heparin—exhibit stronger binding affinities to COX-2 receptor compared to the individual drugs (Figure 3) [26].

This enhanced binding suggests that the complexes introduce additional stabilizing interactions, such as extended hydrogen bonding networks, complementary hydrophobic interactions, or cooperative effects between functional groups of the two components.

Among the complexes, the heparin–diclofenac configuration generally suggests stronger binding than the diclofenac–heparin configuration, reflecting the importance of ligand orientation and the spatial arrangement of functional groups in optimizing receptor interactions. These differences could be tied to how the heparin molecule, as a larger and more flexible entity, aligns itself to maximize interactions with key residues of COX-2 receptor when serving as the ligand.

When comparing these results to those obtained for COX-1, there are clear distinctions in binding behavior. COX-1 and COX-2 share structural similarities, but their active sites have subtle differences that can influence how ligands and complexes interact [24]. COX-2 has a slightly larger and more flexible active site, which may accommodate the bulkier heparin–diclofenac and diclofenac–heparin complexes more effectively, leading to stronger binding compared to COX-1. This structural variability could also account for differences in the binding strengths of individual drugs, such as diclofenac, between the two enzymes.

**Figure 4:**
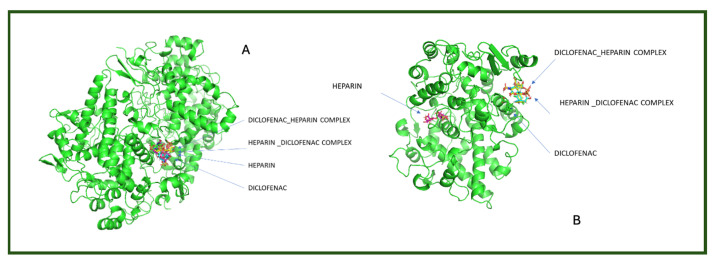
Comparative docking configurations of heparin, diclofenac, and their molecular complexes with the COX-1 receptor (**A**) and COX-2 receptor (**B**), visualized using Chimera [24].

To better visualize the binding interactions within the active sites of the COX-1 and COX-2 receptors, we opened all docking results in the same window using Chimera (Figure 4). This approach allowed for a direct spatial comparison between the different complexes and conformations, offering a clearer understanding of how each ligand or complex aligns and interacts within the receptor’s binding pocket. By overlaying the structures, we were able to observe potential steric hindrances, differences in orientation, and how the presence of two ligands might alter the docking behavior compared to single-ligand configurations (Figure 4).

**Figure 5:**
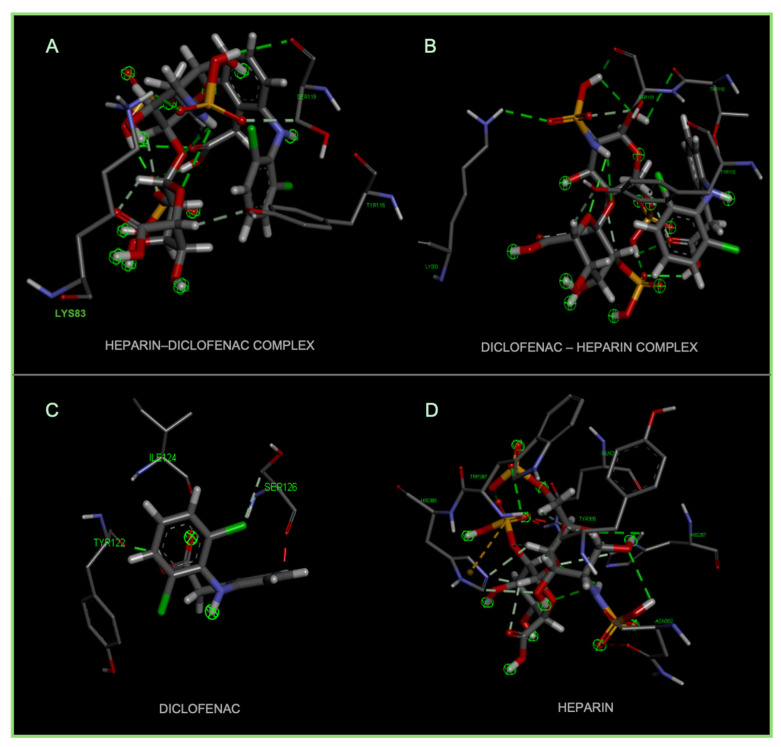
High-resolution 3D docking images of the COX-2 receptor with (**A**) diclofenac–heparin, (**B**) heparin–diclofenac, (**C**) diclofenac, and (**D**) heparin. These images illustrate the spatial orientation of each ligand within the COX-2 active site and highlight the key interacting amino acids involved in binding [25].

It can be observed that for both receptors, the complexes are oriented toward the same binding site, with their spatial arrangements appearing nearly parallel. In the case of the COX-1 receptor, both diclofenac and heparin align within the same active site as their respective complexes, suggesting a shared docking region. However, for the COX-2 receptor, the two individual compounds—diclofenac and heparin—prefer distinct binding sites, indicating a possible difference in binding preferences or steric compatibility (Figure 5). This divergence may reflect structural or conformational differences in the active site architecture between the two isoforms of the cyclooxygenase enzyme.

Figure 5 demonstrates the binding behavior of four different ligand configurations within the COX-2 active site, highlighting how molecular orientation and composition influence interaction patterns. The diclofenac–heparin complex (A) engages key residues such as Arg120 and Tyr355, combining electrostatic and hydrophobic contacts, which may enhance stability and binding affinity compared to single components [26]. Similarly, the heparin–diclofenac complex (B) shows effective anchoring within the active site, with interactions involving the same critical residues, suggesting that the order of conjugation can slightly modulate ligand positioning without drastically altering key contacts.

Diclofenac alone (C) exhibits the classical NSAID binding mode, forming hydrogen bonds with Arg120 and Tyr355 while occupying the hydrophobic pocket, consistent with its known inhibitory activity [27]. In contrast, heparin alone (D) binds primarily through electrostatic interactions with positively charged residues such as Arg120 and Lys83, showing limited hydrophobic engagement, which may reduce overall inhibitory potential.

Together, these results indicate that conjugation of diclofenac with heparin preserves essential interactions while potentially enhancing binding stability and modulating ligand orientation. This insight could inform the design of hybrid or combination molecules with improved efficacy and safety profiles for COX-2 modulation.

These observations underscore the potential of molecular complexes to enhance drug–receptor interactions beyond what is achievable with individual drugs. They also highlight the importance of receptor-specific dynamics in drug design [28]. By leveraging the distinct binding site characteristics of COX-1 and COX-2, it may be possible to tailor therapies that selectively target one enzyme over the other, potentially minimizing side effects and improving therapeutic outcomes. Further investigation into the molecular details of these interactions, such as the specific residues involved and the energetic contributions of different interaction types, would provide deeper insights into the pharmacological implications of these complexes.

To further investigate the interaction modes of the studied systems—diclofenac, heparin, and their respective complexes—with their biological targets, COX-1 and COX-2 (the receptors), we performed a molecular dynamics (MD) simulation in the presence of water as solvent. This step was designed to validate and complement the docking results obtained in the previous stages of the study, by observing the dynamic behavior and potential stability of the complexes under near-physiological conditions.

**Figure 6:**
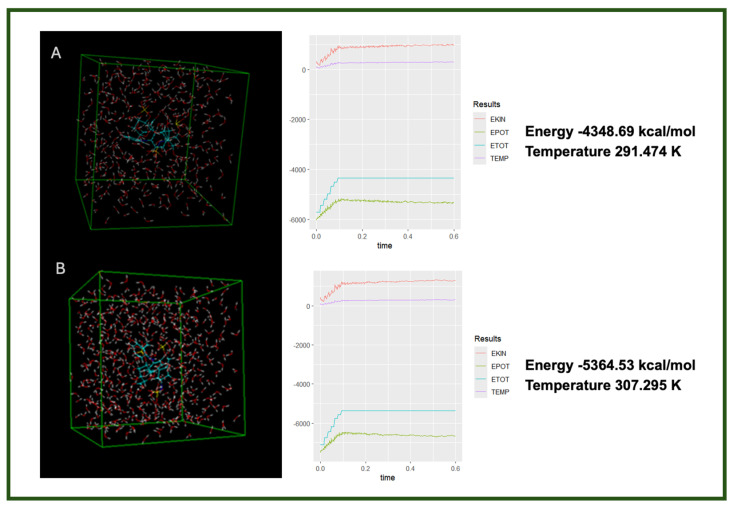
Molecular dynamics simulation results showing the final energy and temperature of two ligand complexes in aqueous solution: (**A**) heparin–diclofenac complex and (**B**) diclofenac–heparin complex [14,29].

The MD simulation was carried out using the HyperChem software package, employing the AMBER force field. A variable temperature setup was used throughout the simulation in order to collect a broader range of data that would contribute to a more accurate prediction of the systems’ structural stability (Figure 6). This approach allows for a more realistic evaluation of the behavior of the complexes over time, under solvent conditions, and offers insight into their potential biological relevance [28].

The data from the molecular dynamics simulations reveal distinct differences in the thermodynamic behavior of the two complexes, despite involving the same components. These differences suggest that the order of interaction—specifically which molecule is treated as the primary ligand—affects the structural arrangement and stabilization of the resulting complex. The lower energy and slightly higher temperature in the heparin–diclofenac system may indicate greater molecular flexibility and stronger intermolecular interactions, such as hydrogen bonding or dipole interactions, allowing for better accommodation within the receptor’s active site or a more relaxed conformation.

In contrast, the diclofenac–heparin complex appears to be more rigid or less interactive, as reflected by the higher energy and lower thermal fluctuations (temperature). This might imply reduced stability or less favorable interaction geometry, potentially affecting its biological relevance. These results highlight the importance of interaction directionality in molecular docking and dynamics, and support the need for dual-orientation studies in complex systems involving large, flexible ligands like heparin.

These computational findings raise the hypothesis that supramolecular association with heparin could, in principle, permit effective COX engagement at lower free diclofenac concentrations. This remains speculative and will require targeted in vitro and in vivo studies (e.g., COX inhibition assays and pharmacodynamic evaluations) to determine whether any dose-sparing or safety advantages occur in practice. All proposed implications regarding potential dose-sparing effects, isoform preferences, or improved safety profiles must be viewed as hypothesis-generating and require rigorous experimental confirmation.

## 4. Clinical Implications and Future Directions

The present in silico findings provide a mechanistic foundation for exploring supramolecular drug design strategies aimed at enhancing the therapeutic performance of nonsteroidal anti-inflammatory agents. The increased binding affinities observed for both COX-1 and COX-2 suggest that pre-formed heparin–diclofenac and diclofenac–heparin complexes may engage the enzyme surface more effectively than either compound alone. Such enhanced binding raises the hypothesis that supramolecular complexation could potentiate COX inhibition, potentially allowing effective blockade at reduced diclofenac concentrations. From a pharmacological standpoint, this implies a possible dose-sparing effect, which if validated, could mitigate the gastrointestinal, renal, and cardiovascular risks associated with chronic NSAID therapy. In light of the well-established cardiovascular safety concerns surrounding diclofenac, future computational and experimental extensions of this approach should evaluate alternative NSAIDs with more favorable safety profiles (e.g., naproxen or ibuprofen) to determine whether similar cooperative interactions can be replicated with clinically safer scaffolds.

From a formulation perspective, the supramolecular complexes described here represent an opportunity for developing localized delivery systems such as topical gels, hydrogels, or transdermal patches. The hydrophilicity and polyanionic character of heparin may promote tissue adherence, local retention, and sustained release, thereby limiting systemic exposure. In addition, molecular complexation may modulate diclofenac’s stability and release kinetics, properties desirable for controlled, site-specific delivery platforms.

A particularly promising translational avenue involves the use of non-anticoagulant heparin derivatives or low-sulfated heparin fragments. These analogs retain the structural features required for supramolecular assembly yet possess minimal anticoagulant activity. Incorporating NAHs into diclofenac formulations could therefore exploit the cooperative interactions identified here without imposing clinically significant bleeding risk. Their demonstrated utility in antiviral and oncologic applications further supports their potential extension to COX-targeted anti-inflammatory strategies.

The results also highlight the critical role of spatial configuration in determining the stability and potential biological relevance of these complexes. Directionality of assembly, whether heparin binds to diclofenac or vice versa, proved decisive in shaping docking energies and thermodynamic behavior. For COX-1, the HD configuration displayed superior stability, whereas for COX-2, the DH orientation was energetically favored. These observations underscore that ligand orientation and assembly sequence might significantly influence functional outcomes, a consideration that should inform both supramolecular drug design and experimental formulation protocols. In practical applications, the molecular ratio and order of assembly may influence biological performance and should therefore be empirically optimized.

The concordance between docking results and molecular dynamics simulations further suggests the consistency of the computational approach. In both COX isoforms, pre-formed complexes show stable interaction patterns in solvated environments, aligning with docking-derived rankings.

Overall, these findings are consistent with the emerging paradigm of supramolecular and multi-target pharmacology, in which therapeutic efficacy is enhanced not solely through highly potent individual agents but by engineering synergistic molecular assemblies with optimized target engagement, stability, and pharmacokinetic control.

However, the present conclusions remain hypothesis-generating and require rigorous experimental validation. The supramolecular complexes examined here are theoretical constructs derived from docking, and we do not demonstrate that such assemblies form in vivo or that a single dominant binding mode exists under physiological conditions. Experimental approaches such as NMR spectroscopy, calorimetry, or biophysical binding assays will be essential to determine whether stable diclofenac–heparin complexes form and whether they modulate COX inhibition.

Finally, our findings rely on legacy docking software (HEX) and a basic implementation of the AMBER force field in HyperChem, which lacks advanced treatments of long-range electrostatics and modern thermostat/barostat algorithms. As such, the present study should be regarded as an exploratory proof-of-concept. Future work using contemporary docking suites (e.g., AutoDock, AutoDock Vina, Glide) and state-of-the-art MD engines (e.g., AMBER with PME electrostatics, GROMACS) will be necessary to obtain a more rigorous structural and energetic characterization of these supramolecular interactions.

## 5. Conclusions

This study provides in silico observations on the molecular interactions of heparin, diclofenac, and their supramolecular complexes with the cyclooxygenase isoenzymes COX-1 and COX-2. Dual-drug molecular docking and molecular dynamics simulations indicate that the heparin–diclofenac and diclofenac–heparin assemblies may display more favorable binding energies than the individual ligands, although these results should be interpreted cautiously given the computational constraints. The simulations suggest that supramolecular complexation can introduce additional stabilizing interactions, which may contribute to the observed trends. In the models examined, the HD configuration tended to show lower docking energies, highlighting the potential influence of ligand orientation and the directionality of molecular assembly. Differences between the COX isoforms, particularly the comparatively more accommodating binding pocket of COX-2, were associated with distinct docking poses, though such findings do not imply functional selectivity. Variations in calculated lipophilicity between the complexes and the parent molecules further illustrate how physicochemical changes can influence predicted receptor interaction. Overall, these results indicate that pre-formed supramolecular complexes may alter ligand–target dynamics, but the conclusions remain provisional. As the work is based exclusively on in silico approaches, using simplified ligand representations and modest simulation parameters, experimental studies will be necessary to determine whether comparable interactions occur under physiological conditions and whether they bear any pharmacological relevance.

## Figures and Tables

**Table 1:** Structure and partition coefficient of the studied compounds [14].

Structure	Compound	logP (Octanol/Water)
Diclofenac	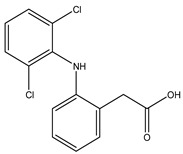	−0.21
Heparin	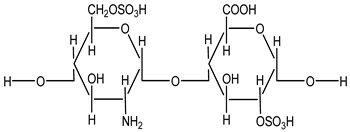	−2.33

**Table 2:** Docking order and docking energies for the supramolecular association of heparin and diclofenac, performed with HEX [15].

Ligand	Receptor	Energy (kcal/mol)
diclofenac	heparin	−140.56
heparin	diclofenac	−146.73

**Table 3:** Bond angles for diclofenac and the two studied complexes, as well as intermolecular distances [14].

Diclofenac	
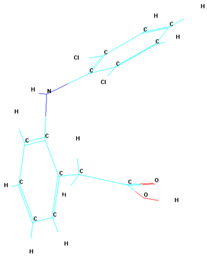	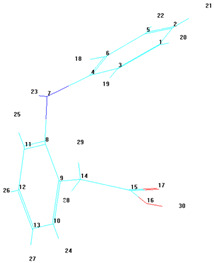
C_4_-N_7_-C_8_ = 118.238^0^	Cl_19_-C_8_ = 3.1115 Å Cl_18_-C_8_ = 4.1203 Å
Diclofenac–heparin complex	
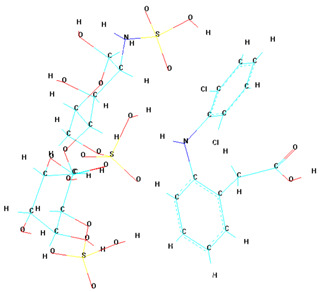	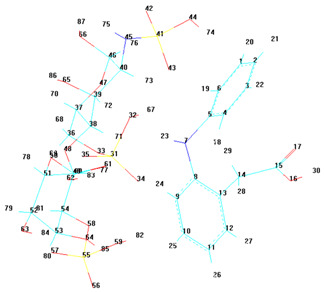
C_4_-N_7_-C_8_ = 117.436^0^	Cl_19_-C_8_ = 4.0900 Å Cl_18_-C_8_ = 3.0933 Å N_45_(heparin)-N_7_(diclofenac) = 5.2282 Å O_48_(heparin)-N_7_(diclofenac) = 4.8945 Å
Heparin–diclofenac complex	
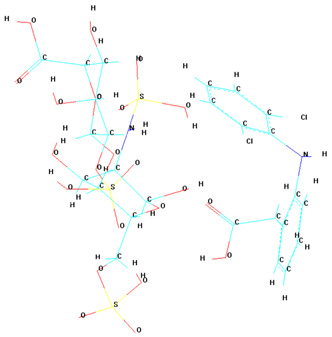	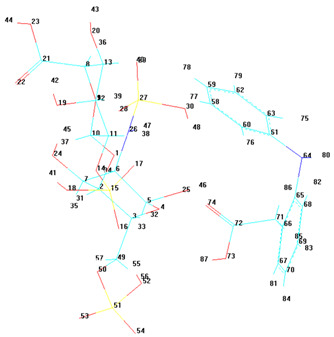
C_61_-N_64_-C_65_ = 117.418^0^	Cl_76_-C_65_ = 3.1381 Å Cl_75_-C_65_ = 4.1213 Å N_26_(heparin)-N_64_(diclofenac) = 7.0725 Å O_1_(heparin)-N_64_(diclofenac) = 6.6219 Å

**Table 4:** Values of physicochemical parameters.

Complex	SA, Å2	V, Å3	µ, D
Diclofenac–Heparin	744.55	1741.33	2.068
Heparin–Diclofenac	773.08	1748.88	4.49

**Table 5:** Partition coefficient of diclofenac–heparin complexes [14].

Compound	logP (Octanol/Water)
Heparin–diclofenac	4.04
Diclofenac–heparin	3.76

**Table 6:** The docking results of the binding energies with COX-1 [15].

Compound	Energy (kcal/mol)
heparin_diclofenac	−358.06
heparin	−309.55
diclofenac	−305.47
diclofenac_heparin	−63.7

**Table 7:** The docking results of the binding energies with COX-2 [14].

Compound	Energy (kcal/mol)
diclofenac_heparin	−468.48
heparin_diclofenac	−434.59
diclofenac	−332.81
heparin	−285.75

## Data Availability

The original contributions presented in this study are included in the article/Supplementary Material. Further inquiries can be directed to the corresponding author.

## Supplementary Materials

The following supporting information can be downloaded at: https://www.mdpi.com/article/10.3390/life15121903/s1, Supramolecular Complex Generation Protocol.

## References

[1] Hirsh J., Levine M.N. (1992). The pharmacology and clinical use of heparin. N. Engl. J. Med..

[2] Garcia D.A., Baglin T.P., Weitz J.I., Samama M.M. (2012). Parenteral anticoagulants: Antithrombotic therapy and prevention of thrombosis, 9th ed.: American College of Chest Physicians Evidence-Based Clinical Practice Guidelines. Chest.

[3] Lever R., Page C.P. (2011). Novel drug development opportunities for heparin. Nat. Rev. Drug Discov..

[4] Altman R., Bosch B., Brune K., Patrignani P., Young C. (2015). Advances in NSAID Development: Evolution of Diclofenac Products Using Pharmaceutical Technology. Drugs.

[5] Rannou F., Pelletier J.P., Martel-Pelletier J. (2016). Efficacy and safety of topical NSAIDs in the management of osteoarthritis: Evidence from real-life setting trials and surveys. Semin. Arthritis Rheum..

[6] Scott L.J., Lamb H.M. (2016). Diclofenac: A review of its use in the management of pain and inflammatory conditions. Drugs.

[7] Moore R.A., Derry S., McQuay H.J. (2015). Single dose oral diclofenac for postoperative pain in adults. Cochrane Database Syst. Rev..

[8] Amzoiu M.-O., Taișescu O., Amzoiu E., Chelu V.A., Popescu S., Rau G., Ciocilteu M.V., Manda C.-V. (2024). Enhancing drug-target interactions through dual-drug docking: A computational study of diclofenac-heparin complexes with factor Xa. J. Sci. Arts.

[9] Kołodziejska J., Kołodziejczyk M. (2018). Diclofenac in the treatment of pain in patients with rheumatic diseases. Reumatologia.

[10] Kaur J., Sanyal S.N. (2011). Diclofenac, a selective COX-2 inhibitor, inhibits DMH-induced colon tumorigenesis through suppression of MCP-1, MIP-1α, and VEGF. Mol. Carcinog..

[11] Bruno R.D.C., Pereira T.V., Saadat P., Rudnicki M., Iskander S.M., Bodmer N.S., Bobos P., Gao L., Kiyomoto H.D., Montezuma T. (2021). Effectiveness and safety of non-steroidal anti-inflammatory drugs and opioid treatment for knee and hip osteoarthritis: Network meta-analysis. BMJ.

[12] Pagadala N.S., Syed K., Tuszynski J. (2017). Software for molecular docking: A review. Biophys. Rev..

[13] Forli S., Huey R., Pique M.E., Sanner M.F., Goodsell D.S., Olson A.J. (2016). Computational protein-ligand docking and virtual drug screening with the AutoDock suite. Nat. Protoc..

[14] HyperChem Professional Release 8. http://www.hyper.com.

[15] https://hex.loria.fr.

[16] https://www.rcsb.org.

[17] Anoaica P.G., Amzoiu E., Bozzini F., Averis L.M.E., Bubulica M.V. (2015). A predictive and invariant “in silico” model for the transmembrane partition coefficient in a wide series of benzene derivatives. Rev. Chim..

[18] Tavares C., Santos T., da Cunha E., Ramalho T. (2023). Molecular Dynamics-Assisted Interaction of Vanadium Complex–AMPK: From Force Field Development to Biological Application for Alzheimer’s Treatment. J. Phys. Chem. B.

[19] Amzoiu E., Amzoiu M.O., Anoaica P.G. (2010). Molecular descriptors for the study of lipophilicity in catecholamine class. Rev. Roum. Chim..

[20] Sidhu R.S., Lee J.Y., Yuan C., Smith W.L. (2010). Comparison of Cyclooxygenase-1 Crystal Structures: Cross-Talk between Monomers Comprising Cyclooxygenase-1 Homodimers. Biochemistry.

[21] Goodman M.C., Xu S., Rouzer C.A., Banerjee S., Ghebreselasie K., Migliore M., Piomelli D., Marnett L.J. (2018). Dual cyclooxygenase-fatty acid amide hydrolase inhibitor exploits novel binding interactions in the cyclooxygenase active site. J. Biological. Chem..

[22] Amzoiu D., Stoian A.M., Amzoiu E., Rau G. (2015). Study concerning the inhibitory activity upon myeloperoxidase of some oxicam class derivatives using the docking molecular technique. Rev. Chim..

[23] Amzoiu M., Popescu S., Amzoiu E., Chelu A., Ciocilteu M.-V. (2024). The docking study of the interaction between food supplements and binimetinib. J. Sci. Arts.

[24] Pettersen E.F., Goddard T.D., Huang C.C., Couch G.S., Greenblatt D.M., Meng E.C., Ferrin T.E. (2004). UCSF Chimera–A visualization system for exploratory research and analysis. J. Comput. Chem..

[25] Biovia D., Berman H., Westbrook J., Feng Z., Gilliland G., Bhat T., Richmond T.J. (2016). Dassault Systèmes BIOVIA, Discovery Studio Visualizer.

[26] Amzoiu M.-O., Popescu G.-S., Amzoiu E., Ciocîlteu M.-V., Manda C.V., Rau G., Gresita A., Taisescu O. (2025). Modulatory effects of caffeine on imatinib binding: A molecular docking study targeting CYP3A4. Life.

[27] Amzoiu M., Chelu A., Popescu S., Amzoiu E., Ciocilteu M. (2023). Interaction between food supplements and drugs using molecular docking. J. Sci. Arts.

[28] Amzoiu M., Amzoiu E., Belu I., Popescu S., Cheita G., Amzoiu D. (2019). Identification of molecular fragments responsible for the antimicrobial activity of acetamide derivatives. J. Sci. Arts.

[29] R Core Team (2021). R: A Language and Environment for Statistical Computing.

