# Supplementary material for: Molecular Docking Analysis of Heparin–Diclofenac Complexes: Insights into Enhanced Cox Enzyme Inhibition for Pain Management
Source: Life (Basel). 2025 Dec 12;15(12):1903. doi: 10.3390/life15121903 (PMC12734184; doi:10.3390/life15121903)
Supplement: Supplementary file 1 [file life-15-01903-s001.zip › life-4004126-supplementary.pdf]

SUPRAMOLECULAR COMPLEX GENERATION PROTOCOL

1. Molecular sketching and 3D structure generation

Diclofenac and the heparin fragment were first drawn in 2D using HyperChem Professional (Release 8.0). The software was then used to automatically generate the corresponding 3D geometries. Each structure was subjected to geometry optimization. The optimized molecular structures were subsequently saved in Protein Data Bank (.pdb) format for compatibility with downstream docking applications [14].

2. Preparation of files for docking

The resulting PDB files were transferred to the working directory of HEX 8.0.0 (C:/Program Files/Hex 8.0/examples), which enables HEX to recognize and process the input structures for docking simulations [15].

3. Docking diclofenac (receptor) with heparin (ligand): diclofenac_heparin complex

Diclofenac was loaded as the receptor and heparin as the ligand. Docking calculations were performed using the "Shape + Electro" correlation mode in HEX. For each run, the Top 100 docking poses were generated and ranked according to docking energy. The most stable and sterically plausible pose was selected and saved as diclofenac_heparin.pdb [23].

4. Docking heparin (receptor) with diclofenac (ligand): heparin_diclofenac complex

In a reciprocal docking simulation, heparin was defined as the receptor and diclofenac as the ligand. The same docking parameters ("Shape + Electro" correlation and Top 100 solutions) were used to ensure methodological consistency. The optimal pose was selected using the same energetic and geometric criteria and saved as heparin_diclofenac.pdb [26].

References:

14 HyperChem Professional Release 8; Hypercube, Gainesville, FL, USA.

15 Ritchie, D.W. HEX 8.0.0: Protein docking software. Available online: https://hex.loria.fr

23 Amzoiu, M.; Popescu, S.; Amzoiu, E.; Chelu, A.; Ciocilteu, M.-V. The docking study of the interaction between food supplements and binimetinib. Journal of Science and Arts 2024, 24(2), 419–428.

26 Amzoiu, M.-O.; Popescu, G.-S.; Amzoiu, E.; Ciocîlteu, M.-V.; Manda, C.V.; Rau, G.; Gresita, A.; Taisescu, O. Modulatory effects of caffeine on imatinib binding: a molecular docking study targeting CYP3A4. Life 2025, 15, 1247. https://doi.org/10.3390/life15081247
